# A Deterministic Model for Estimating Indoor Radon Concentrations in South Korea

**DOI:** 10.3390/ijerph16183424

**Published:** 2019-09-15

**Authors:** Ji Hyun Park, Cheol Min Lee, Dae Ryong Kang

**Affiliations:** 1 Department of Mathematics, Ajou University, Suwon 16499, Korea; jhn1105@gmail.com; 2 Department of Chemical and Biological Engineering, SeoKyeong University, Seoul 02713, Korea; cheolminlee1@gmail.com; 3 Department of Precision Medicine & Biostatistics, Wonju College of Medicine, Yonsei University, Wonju 26426, Korea

**Keywords:** indoor radon, mean annual radon concentrations, mass balance equation, geographical factor, building characteristics, meteorological factor, South Korea

## Abstract

Estimating long-term exposure to indoor radon is necessary to determine the effects of indoor radon exposure on health. However, measuring long-term exposure to radon is labor intensive and costly. While developing models for estimating indoor radon concentrations are very difficult and unrealistic due to the many factors affecting radon concentrations, several studies have attempted to estimate indoor radon concentrations with mathematical models based on mass balance equations. However, these models are only applicable to specific regions or situations, and some require actual measurement data. This study sought to develop a widely applicable model for estimating mean annual indoor radon concentrations in actual residences considering seasonal variations in indoor radon. The model is based on a mass balance equation using data on geographical factors, building characteristics, meteorological factors, and nationwide radon surveys. The primary factor in our model is the infiltration factor, which can vary according to region, building materials, cracks, floor type, etc. In this study, infiltration factor was calculated according to the type of housing and groundwater usage, and the results thereof were applied to estimate indoor radon concentrations. Overall, measured concentrations and estimates of indoor radon concentrations using the infiltration factor were similar. This model showed better performance than our previous model, except for a few high concentration residences.

## 1. Introduction

Radon is a radioactive, colorless, odorless and tasteless noble gas, known to be a major component of natural radiation. The accumulation of radon and its decay products in the lungs can cause lung cancer and is the second leading cause of lung cancer after smoking [1,2]. In indoor environments where people spend a lot of time, such as their residence, long-term exposure to radon poses a considerable health risk [3]. For estimating long-term exposure to radon indoors, information on indoor radon levels and indoor residence time is needed. However, measuring indoor radon concentrations is labor intensive and costly, limiting studies of the adverse health effects related to long-term indoor radon exposure. To overcome these problems, several studies have sought to estimate indoor radon concentrations with mathematical models based on mass balance equations. These models and applications were previously reviewed by Park et al. [4] who found them to be applicable only to specific regions or situations or to require actual measurement data. For Korea, Park et al. [5] developed a model for estimating indoor radon concentrations using data from surveys between January and April 2016. While this model offered wider application for a number of residences, not to a specific site, and could be used in situations where actual measurements for input variables were lacking, the model was only of use for estimating indoor radon concentrations for detached houses.

The aim of this study was to develop a more widely applicable model for estimating indoor radon concentrations for a greater number of residential types. To do so, we focused on constructing a model based on mass balance equations, using data on geographical factors, building characteristics, meteorological factors, and nationwide radon surveys. We also considered seasonal variations in indoor radon concentrations, which are high in the winter and low in the summer. The main component of our model is the infiltration factor, which can vary according to building characteristics (e.g., region, building materials, cracks, and floors). Estimating infiltration factor values according to type of housing and groundwater usage, we applied them to estimate indoor radon concentrations, which we compared to actual measured radon concentrations.

## 2. Materials and Methods

### 2.1. Indoor Radon Concentrations and Questionnaires

Between October 2015 and December 2018, indoor radon concentrations at 1437 residences were collected using passive alpha-track detectors (Raduet Model RSV-8, Radosys Ltd., Budapest, Hungary) over a minimum of 90 days. The radon concentrations were measured at two points in each residence, and the points were selected from spaces where residents primarily spend most of their time, such as living rooms and bedrooms. The geometric means of radon concentrations from the two points within the residences were used to develop a model of radon concentrations: because of the log-normal distribution of radon concentrations, log transformation was applied. Questionnaires were also administered to collect information on address (city/province and city/county/district), type of house, building materials, the location and number of cracks in the building, usage of groundwater, building materials for the walls, and resident’s ventilation habits. In total, 1390 residences completed the questionnaire without any missing items. Finally, 47 selected outliers were removed from the log-transformed radon data for the stability of the developed model, and 1343 residences were used to develop the model. Since indoor radon concentrations were measured regardless of season, data obtained from radon surveys were standardized to eliminate seasonal variations. To do so, indoor radon concentrations were converted to annual mean radon concentrations in homes using the correction factors described by Park et al. [6]. Table 1 summarizes the annual mean radon concentrations according to residential type and groundwater usage.

### 2.2. Nationwide Radon Survey Data

For model development, we also used nationwide radon survey data from the National Institute of Environmental Research, which conducted three surveys in the winter months from 2011 to 2016 in Korea [7]. The surveys in 2011–2012, 2013–2014, and 2015–2016 were conducted for about 11,000, 8000 and 12,000 residences, respectively. The surveyed houses were selected considering the type of residential structure, such as detached houses, apartments, and multi-family dwellings. In apartments and multi-story buildings, only residences on the third floor or below were selected. About 14,152 radon values measured at detached houses were extracted, and 13,975 were used after removing selected outliers after log-transformation (AM = 116.71 ± 103.43 ${\mathrm{Bq}\;m}^{-3}$ and GM = 86.52 ± 2.14 ${\mathrm{Bq}\;m}^{-3}$).

### 2.3. Geographical and Meteorological Data

Using a structural equation model, Lee et al. [8] showed that a high greenery ratio significantly increases indoor radon concentrations. The Environmental Geographic Information Service maintained by the Ministry of Environment in Korea provides access to greenery ratio data, which reflect the area of green area compared to administrative regions according to the city/county/district [9]. Green areas comprise forest and grassland areas and do not include agricultural spaces, such as rice fields.

Ventilation is also well known as a major factor affecting indoor radon concentrations [10], and ventilation rates are known to be affected by meteorological factors, such as indoor and outdoor temperature differences and wind speeds. For smoothness of annual deviations in monthly average meteorological factors, we used 30-year averages (1981–2010) from the National Climate Data Service System [11].

### 2.4. Mass Balance Equation of Indoor Radon

Since indoor radon is affected by several parameters, a large number of parameters would be needed to develop a model. However, we focused only on factors shown to affect indoor radon concentrations in previous studies, such as soil, building materials, outdoor air, and ventilation. In Park et al. [5], the following system of differential equations for estimating indoor radon concentrations was presented based on a mass balance equation by modifying a model suggested by Font et al. [12] and Arvela et al. [13] as follows:

(1) $$\begin{array}{c} \frac{dC_{i}}{dt}=\left\{k_{d,s}\left(C_{s}-C_{i}\right)+k_{a}\Delta P_{s-i}C_{s}\right\}\frac{S_{g}}{V}+k_{d,bm}\left(C_{bm}-C_{i}\right)\frac{S_{bm}}{V}-\lambda_{v}\left(C_{i}-C_{o}\right)-\lambda C_{i},\\ \frac{dC_{s}}{dt}=E_{s}\frac{S_{g}}{V}-\left\{k_{d,s}\left(C_{s}-C_{i}\right)+k_{a}\Delta P_{s-i}C_{s}\right\}\frac{S_{g}}{V}-\lambda C_{s},\\ \frac{dC_{bm}}{dt}=E_{bm}\frac{S_{bm}}{V}-k_{d,bm}\left(C_{bm}-C_{i}\right)\frac{S_{bm}}{V}-\lambda C_{bm}, \end{array}$$

(2) $$\lambda_{v}=(f_{t}\left|T_{i}-T_{o}\right|+f_{w}u^{2})\sqrt{N},$$

where $C_{bm}$ = radon concentrations in building materials $\left({\mathrm{Bq}\;m}^{-3}\right)$, $C_{i}$ = indoor radon concentrations $\left({\mathrm{Bq}\;m}^{-3}\right)$, $C_{s}$ = radon concentrations in soil $\left({\mathrm{Bq}\;m}^{-3}\right)$, $C_{o}$ = radon concentrations in outdoor air $\left({\mathrm{Bq}\;m}^{-3}\right)$, $E_{bm}$ = effective radon exhalation rate of building materials $\left({\mathrm{Bq}\;m}^{-2}h^{-1}\right)$, $E_{s}$ = effective radon exhalation rate of soil $\left({\mathrm{Bq}\;m}^{-2}{\;h}^{-1}\right)$, $f_{t},f_{w}$ = fitting parameters, $k_{a}$ = advection transfer coefficient of soil $\left({m\;\mathrm{Pa}}^{-1}\;h^{-1}\right)$, $k_{d,bm}$ = diffusion transfer coefficient of building materials $\left({m\;h}^{-1}\right)$, $k_{d,s}$ = diffusion transfer coefficient of soil $\left({m\;h}^{-1}\right)$, N = the number of ventilations (dimensionless),$S_{bm}$ = indoor surf‘ace area of radon containing building materials $\left(m^{2}\right)$, $S_{g}$ = building area towards the ground $\left(m^{2}\right)$, $T_{i},T_{o}$ = indoor and outdoor temperatures, respectively (℃), u = wind speed $\left(m\;s^{-1}\right)$, V = volume of the indoors $\left(m^{3}\right)$,  λ = radon decay constant $\left(h^{-1}\right)$, $\lambda_{v}$ = ventilation rate $\left(h^{-1}\right)$, and $\Delta P_{s-i}$ = soil-indoor pressure difference (Pa).

At a steady state, we obtain the following equations:

(3) $$\begin{array}{c} C_{i}=\frac{E_{s}\frac{S_{g}}{V}+E_{bm}\frac{S_{bm}}{V}+\lambda_{v}C_{o}-\lambda \left(C_{s}+C_{bm}\right)}{\lambda +\lambda_{v}},\\ \lambda_{v}=(f_{t}\left|T_{i}-T_{o}\right|+f_{w}u^{2})\sqrt{N}. \end{array}$$

In Equation (3), $\frac{E_{s}S_{g}}{V}+\frac{E_{bm}S_{bm}}{V}$ represents entrance concentration rates from the soil and building materials. However, information on all parameters in the equation is required to calculate radon exhalation rates from the soil and building materials, and since available information is often limited, this approach is unrealistic. For this reason, we chose to construct a statistical model with which to estimate $\frac{E_{s}S_{g}}{V}+\frac{E_{bm}S_{bm}}{V}$.

### 2.5. Infiltration Factor Model

We denoted $\frac{E_{s}S_{g}}{V}+\frac{E_{bm}S_{bm}}{V}$ in Equation (3) as S, the infiltration factor, representing entrance concentration rates from the soil and building materials. Based on the mass balance equation and input variables described above, we can calculate S directly using the following equation:

(4) $$\begin{array}{c} S=E_{s}\frac{S_{g}}{V}+E_{bm}\frac{S_{bm}}{V}=C_{i}\left(\lambda +\lambda_{v}\right)+\lambda \left(C_{s}+C_{bm}\right)-\lambda_{v}C_{o},\\ \lambda_{v}=(f_{t}\left|T_{i}-T_{o}\right|+f_{w}u^{2})\sqrt{N}. \end{array}$$

Meanwhile, entrance concentration rates from soil and building materials are also affected by geographical characteristics and building design. Greenery ratio can be considered a factor connected to the exhalation rate of radon from surfaces [8]; cracks in the building and floors may also be factors. Therefore, we may consider the following statistical model for estimating entrance concentration rates from the soil and building materials for residence i:

(5) $$S_{i}=\beta_{0}+\beta_{1}X_{1i}+\beta_{2}X_{2i}+\beta_{3}Y_{1i}+\beta_{4}Y_{2i}+\beta_{5}Y_{3i}+\epsilon_{i,}$$

where $S_{i}$ = entrance concentration rates from soil and building materials for each residence i, $X_{1i}=$ greenery ratio of the administrative district to which residence i belongs, $X_{2i}=$ geometric mean of indoor radon levels of the administrative district to which residence i belongs, $Y_{1i}=$ type of building material of residence i, $Y_{2i}=$ degree of cracks in residence i, $Y_{3i}=$ floors of residence i, and $\epsilon_{i}=$ error term of residence i.

### 2.6. Model Application

Since our model hinges on the infiltration factor, we need to obtain values for entrance concentration rates from soil and building materials for individual residences. To do so, we calculate S using Equation (4) and use it to estimate the parameters in Equation (5). As shown in Table 1, since indoor radon concentrations in detached houses are higher than those in other residences, the factors that need to be considered depend on the type of residence. While radon in groundwater is known to influence indoor radon concentrations [14], information about radon in groundwater is lacking in Korea. To consider the influence of type of residence and groundwater on indoor radon concentrations, we estimated the infiltration factor S by dividing the model into four types according to the type of residence and usage of groundwater. Thus, we incorporated Equations (3) and (4) and S in Equation (5) to predict indoor radon concentrations $C_{i}$. Finally, we evaluated the predictive abilities of all four model types.

Input variables for calculating S in Equation (4) and parameters for estimating S in Equation (5) are as follows:

(1) As mentioned above, indoor radon concentrations were converted to annual mean radon concentrations in residences using the correction factors in Park et al. [6].
(2) Since radon entry from building materials is relatively small, compared to that from soil [15], we assumed that $C_{s}+C_{bm}\approx C_{s}$. Furthermore, since information on radon concentrations in soil in Korea is lacking and radon concentrations in soil are not measured under the same conditions, they are not suitable for use as a representative value of $C_{s}$. In Korea, indoor radon concentrations are high in granite zones, compared to other areas [16]. The nationwide radon survey data for measured radon concentrations that we used in this study were obtained only during the winter season, reducing the impact of factors such as ventilation thereon. Therefore, using the data obtained from nationwide radon surveys, we estimated radon concentrations in soil for 233 administrative districts (city/county/district) by assigning weights. The weight was calculated as the ratio of regional geometric mean to total geometric mean. In the case of radon concentrations in outdoor air $C_{o}$, we estimated radon concentrations in outdoor air for 17 administrative districts (city/province) in the same manner as above.
(3) Since ventilation rates are affected by meteorological factors, such as indoor and outdoor temperature differences and wind speeds [10], mean monthly wind speed and outdoor temperature were used to calculate ventilation rate. For smoothness of annual deviations in the mean monthly meteorological factors, we used 30-year averages (1981–2010). Moreover, for calculating ventilation rate $\lambda_{v}$, the values of fitting parameters $f_{t}$ and $f_{w}$ were assumed to be the same as those in Park et al. [5]. Additionally, weights were assigned to N differently according to the resident’s ventilation habits.
(4) The radon decay constant is $7.56\times 10^{-3}{\;h}^{-1}$ [17].

## 3. Results and Discussion

### Results of Parameter Estimation

**Figure 1:**
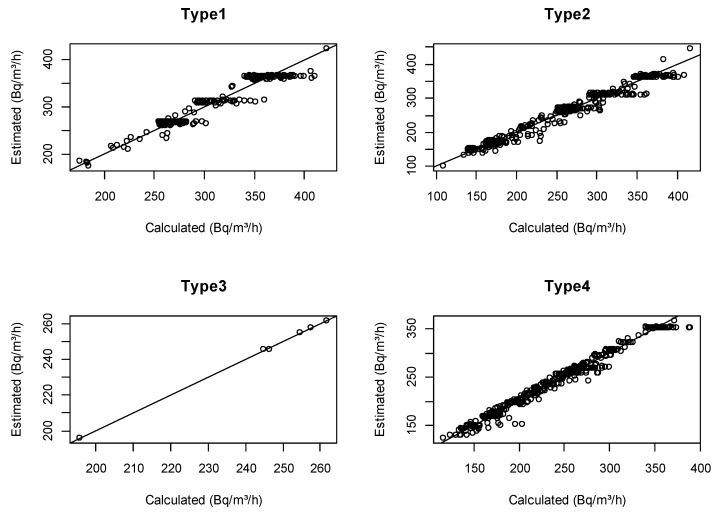
Comparison of calculated and estimated values of infiltration factor S. according to residence characteristics.

Table 2 shows the estimates of the coefficients of the infiltration factor model (R version 3.5.1). Therein, the influences of geographical parameters, such as greenery ratio and geometric means of indoor radon levels of administrative districts, were found to be statistically significant (p<0.05), while the influence of building materials was not (p>0.05). As the number of cracks increased, infiltration factor values also increased, although differences were not statistically significant (p>0.05). Also, the higher the number of floors, the higher infiltration factor values were, although differences in these values were statistically significant only for detached houses (p<0.05). Meanwhile, calculated ventilation rates varied from 0.24 to 0.73 $h^{-1}$ (AM = 0.34 ± 0.08 $h^{-1}$), and these values were included to calculate infiltration rate, causing it to vary from 0.1 to 1 $h^{-1}$ [18]. With these results, the infiltration factor model was applied to estimate entrance concentration rates from soil and building materials. In order to evaluate the appropriateness of the model, the calculated values of S using Equation (4) and the estimated values of S using Equation (5) were compared (Figure 1). The model performed better for residences other than detached houses, although calculated and estimated infiltration factor values followed similar trends overall.

**Figure 2:**
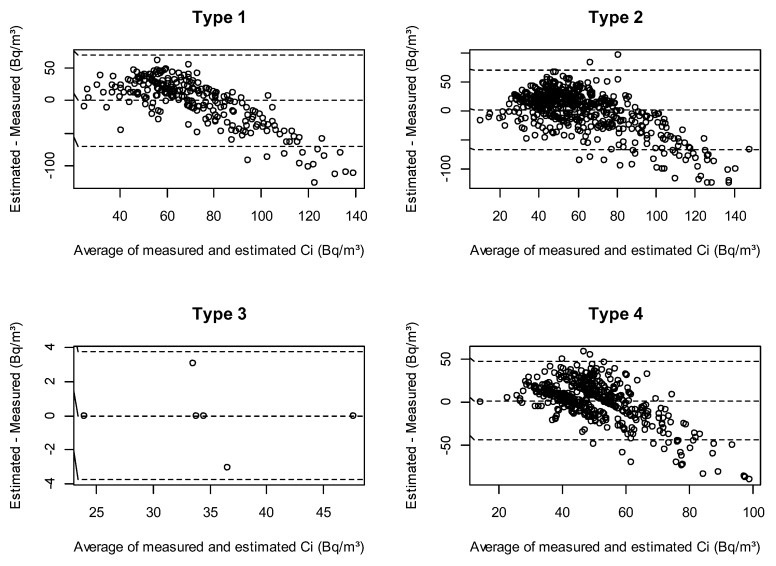
Comparisons of calculated and estimated values of indoor radon concentrations according to residence characteristics.

With the infiltration factor S estimated in Equation (5), we predicted indoor radon concentrations using Equation (3). Figure 2 shows the agreement between the measured and estimated indoor radon concentrations according to residence characteristics. Since the results indicated better performance for residences other than detached houses in the infiltration factor model, better agreement was also obtained for these residences when estimating indoor radon concentrations. For Type 3, the number of residences was small, but the variance was small, showing the best performance. Our model did not show better performance for a few high concentration residences.

## 4. Conclusions

This study was conducted as part of an increased effort to assess the effects of indoor radon exposure on health in Korea. Through further research, our final aims are to develop a model with which to estimate cumulative exposure to radon and establish its relationship with lung cancer. Although developing a model for estimating indoor radon concentrations is difficult and seemingly unrealistic due to the many factors affecting indoor concentrations, doing so is necessary to determine the effects of long-term exposure to radon indoors. Although Park et al. [5] developed the first model for estimating indoor radon concentrations in Korea when actual measurements for input variables were not available, the applicability of the model was limited. In this paper, we constructed a model for estimating indoor radon concentrations considering geographical characteristics, building characteristics, meteorological characteristics, and resident behavior, and the applicability and performance of this model has been improved over the previous one suggested by Park et al. [5].

This study had some limitations. Although the performance of the present model is better than that of previous models, our model did not show better performance for a few high concentration residences. This is likely because about 88% of the residences surveyed in our study had measured radon concentrations less than 100 $\mathrm{Bq}\cdot m^{-3}$. If indoor radon levels were more evenly distributed across low-concentration and high-concentration residences, the model performance may have been better. Another limitation is related to the factors affecting indoor radon levels. Ventilation rate, which is an important factor affecting indoor radon levels, was estimated by relying on ventilation habits reported in questionnaires without actual measurement. Thus, incorrect resident responses could lead to large differences in measured and estimated radon concentrations. Therefore, the accuracy of our model could be expected to improve if other parameters that can be used to estimate ventilation rate are considered, rather than responses on a questionnaire. Moreover, other building characteristics, such as construction year, can also be relevant factors. Therefore, the performance of our model in future studies could be better by considering more detailed information on ventilation, as well as other building characteristics. Finally, estimating cumulative exposure to indoor radon is necessary to confirm a relationship between residential radon and lung cancer. According to an UNSCEAR report [3], annual effective doses of radon are to be calculated as follows:

E=Q×F×T×K

where Q is the mean annual radon concentration, F is the equilibrium factor between indoor radon and its decay product, T is the annual residential time, and K is the dose conversion coefficient.

Our model for estimating indoor radon concentration is expected to be an important tool for estimating long-term exposure to radon and assessing the effects of indoor radon exposure on health in Korea.

## Figures and Tables

**Table 1:** Summary of annual mean radon concentrations according to residential type and groundwater usage.

	Type of Residence	Groundwater Usage	${\mathrm{AM}}^{1}\pm {\mathrm{SD}}^{2}\;(Bq\;\cdot \;m^{-3})$	${\mathrm{GM}}^{3}\pm {\mathrm{GSD}}^{4}\;(Bq\;\cdot \;m^{-3})$
Type 1	Detached house	Yes	71.07 ± 38.05	62.19 ± 1.68
Type 2		No	58.98 ± 37.27	49.71 ± 1.78
Type 3	Other residences	Yes	34.98 ± 7.76	34.26 ± 1.25
Type 4		No	48.73 ± 22.05	44.48 ± 1.53
Total			57.62 ± 33.75	49.86 ± 1.70

^1^ Arithmetic mean, ^2^ standard deviation, ^3^ geometric mean, ^4^ geometric standard deviation.

**Table 2:** Estimates of the coefficients of the infiltration factor model according to residence characteristics.

Predictor Variables	Multiple Linear Regression Model							
	Type 1 ^1^		Type 2 ^2^		Type 3 ^3^		Type 4 ^4^	
	$\beta \;\left(SE{\;}^{5}\right)$	*p-*Value	$\beta \;\left(SE{\;}^{5}\right)$	*p-*Value	$\beta \;\left(SE{\;}^{5}\right)$	*p-*Value	$\beta \;\left(SE{\;}^{5}\right)$	*p-*Value
(Intercept)	−15.89 (6.05)	0.009	−8.90 (2.58)	0.001	4.49 (5.77)	0.579	11.09 (2.11)	<0.001
$\mathrm{Greenery}\;\mathrm{ratio}\;\left(X_{1}\right)$	0.18 (0.07)	0.015	0.12 (0.03)	<0.001	−0.10 (0.04)	0.242	0.08 (0.03)	0.002
GM ^6^ of indoor radon levels of administrative district $\left(X_{2}\right)$	2.73 (0.07)	<0.001	2.72 (0.02)	<0.001	2.59 (0.07)	0.018	2.48 (0.02)	<0.001
Building materials								
Concrete	Ref.		Ref.		Ref.		Ref.	
Red brick	−1.52 (3.00)	0.613	−2.62 (1.37)	0.055	−4.00 (1.68)	0.253	1.29 (1.71)	0.449
Cement block	−0.41 (2.96)	0.890	−4.76 (1.43)	0.001	−6.88 (1.68)	0.152	−2.81 (3.29)	0.394
Soil	−2.54 (3.98)	0.524	−0.22 (2.81)	0.938				
Wood	−8.01 (5.48)	0.145	−5.32 (2.75)	0.054				
Others	−3.22 (3.52)	0.361	−7.16 (2.06)	0.001			−14.19 (9.13)	0.121
$\mathrm{Number}\;\mathrm{of}\;\mathrm{crack}\;\mathrm{locations}\;\left(Y_{2}\right)$								
0–1	Ref.		Ref.				Ref.	
≥2	1.17 (2.44)	0.632	2.43 (1.64)	0.139			2.24 (2.58)	0.384
$\mathrm{Floors}\;\left(Y_{3}\right)$								
≤1	Ref.		Ref.				Ref.	
≥2	−4.94 (2.34)	0.036	−5.56 (1.35)	<0.001			−0.58 (1.49)	0.697

^1^ Detached house and groundwater use, ^2^ detached house and no groundwater use, ^3^ other residences and groundwater use, ^4^ other residences and no groundwater use, ^5^ standard error, ^6^ geometric mean.
